# Electrochemical and passivation behavior investigation of ferritic stainless steel in simulated concrete pore media

**DOI:** 10.1016/j.dib.2015.08.016

**Published:** 2015-09-01

**Authors:** Hong Luo, Huaizhi Su, Chaofang Dong, Kui Xiao, Xiaogang Li

**Affiliations:** a College of Mechanics and Materials, Hohai University, Nanjing 210098, China; b State Key Laboratory of Hydrology-Water Resources and Hydraulic Engineering, Hohai University, Nanjing 210098, China; c Institute of Advanced Materials and Technology, University of Science and Technology Beijing, Beijing 100083, China

**Keywords:** Ferritic stainless steel, AES, Concrete, Passive film

## Abstract

The applications of stainless steel are one of the most reliable solutions in concrete structures to reduce chloride-induced corrosion problems and increase the structures service life, however, due to high prices of nickel, especially in many civil engineering projects, the austenitic stainless steel is replaced by the ferritic stainless steels. Compared with austenite stainless steel, the ferritic stainless steel is known to be extremely resistant of stress corrosion cracking and other properties. The good corrosion resistance of the stainless steel is due to the formation of passive film. While, there is little literature about the electrochemical and passive behavior of ferritic stainless steel in the concrete environments. So, here, we present the several corrosion testing methods, such as the potentiodynamic measurements, EIS and Mott–Schottky approach, and the surface analysis methods like XPS and AES to display the passivation behavior of 430 ferritic stainless steel in alkaline solution with the presence of chloride ions. These research results illustrated a simple and facile approach for studying the electrochemical and passivation behavior of stainless steel in the concrete pore environments.

## Specifications table

**Table:** 

Subject area	Material science
More specific subject area	Corrosion science
Type of data	Table, image
How data was acquired	Electrochemical workstation, X-ray photoelectron spectroscopy, Auger Electronic Spectrometer
Data format	Raw, analyzed
Experimental factors	Environment, potential
Experimental features	Artificial passive film growth
Data source location	Hohai University, Nanjing, China
Data accessibility	Data are available here with this article

## Value of the data

• The immersion time plays important roles in evolution of passive film composition and corrosion resistance.
• The passive film of ferritic stainless steel presenting a thinner n-type layer in the simulated concrete pore environments and its primary constituents of passive film is (Cr, Fe)-oxides, however, there is no chloride ions incorporation into the passive film.
• The data presented here can be used to characterize the passive behavior and passive film composition of ferritic stainless steel in the concrete environments.

## 1 Data, experimental design, materials and methods [1]

### 1.1 Materials and solutions

Here, the testing specimens were cut from a sheet of 430 ferritic stainless steel with the thickness of 5 cm. The nominal chemical composition (wt%) of the stainless steel were as follows: C 0.035, Si 0.50,Cr 17.30, Ni 0.14, Mn 0.35, S 0.004, P 0.029 and Fe balance. The specimens size were 1 cm×1 cm, then ground sequentially from 400 to 2000 grit SiC paper, polished with 0.1 μm alumina powder, degreased with alcohol and deionised water, then, dried in cooling air. The testing solution is the saturated calcium hydroxide solution, it were prepared to simulate the electrolytes contained in the pores of concrete environment, meanwhile, the 1% sodium chloride was added in to the solution, to observe the effect of chloride on its passive film. All the solutions were prepared with double distilled water, and the pH value of testing solution was 13, which was regularly checked using a Mettler pH meter. More important, the solutions were used immediately after preparation to avoid carbonation effects and all reagents were of at least ACS grade.

## 2 Electrochemical measurements and surface characterization

### 2.1 Electrochemical behavior measurements

The electrochemical measurements were performed by using the PAR 2273 electrochemical workstation at ambient temperature (25 °C). The standard three electrodes cell corrosion testing system, a platinum foil and a saturated calomel electrode (SCE), which connected to the cell via a Luggin capillary, were used as the counter and reference electrodes, respectively. Before test, the solution was purged with pure nitrogen for 1 h, and the gas flow was maintained during the whole testing. Prior to electrochemical experiment, samples were initially reduced potentiostatically at -0.8 V for 20 min to remove air-formed oxides. The electrochemical impedance spectroscopy (EIS) measurement started after the stable of open-circuit potential (OCP), the frequency of EIS was swept from 100 kHz down to 10 mHz, at 10 data cycles/decade, with the applied AC amplitude of 10 mV. The impedance spectra were collected for increasing immersion times from 12 h to 480 h, and the impedance data were fitted by Zsimpwin software. The capacitance test was carried out at a fixed frequency of 1 kHz during a 50 mV/Step in the potential range from −1.0 to 1.2 V (vs. SCE). Potentiodynamic polarization tests were carried out using a scan rate of 1.0 mV s^−1^ starting from −0.6 V vs. OCP to transpassive potential. Each experiment was repeated several times under the same conditions to control the reproducibility, keeping the error of several fitting parameters less than 5%.

### 2.2 The AES and XPS characterization

Before the experiments, the surface pre-treatment is very important. A negative potential was applied to remove the surface oxide layer, and then immersion in the solution for 3 h at OCP in order to form of a stationary passive film. The AES depth profiles were measured with a scanning Auger microprobe with a coaxial electron gun and a cylindrical mirror analyzer. The sputtering rate, as determined on a thermal oxidation SiO_2_/Si standard, was approximately 2 nm/min. The chemical composition of the passive film was investigated by XPS with a monochromatic Al K_α_ radiation source and a hemispherical electron analyzer operating at the pass energy of 25 eV. The curve fitting was performed by the commercial software Xpspeak version, which contained the Shirley background subtraction and Gaussian–Lorentzian tail function for better spectra fitting.

## 3 The electrochemical behavior of ferritic stainless steel in the simulated concrete media

### 3.1 The passivation behavior of ferritic stainless steel

**Fig. 1:**
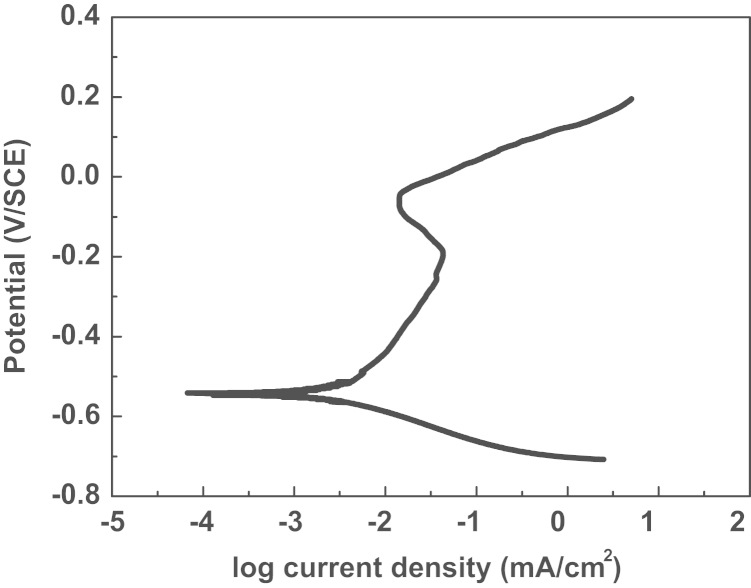
Potentiodynamic polarization curve of 430 ferritic stainless steel in the saturated calcium hydroxide solution with the presence of chloride ions at the scanning rate 0.5 mV/S.

The anodic potentiodynamic curves can provide some important features concern with the electrochemical behavior of stainless steel in certain environments. Fig. 1 shows the potentiodynamic polarization curves of 430 ferritic stainless steel in alkaline solution. It can be seen that ferritic stainless steel displays typical passive behavior, the curves pass through active region, active passive transition region, passive region and transpassive region with increasing potential from free corrosion potential to anode direction. As shown in the Fig. 1, the passive region of 430 ferritic stainless steel in alkaline solution is from about the −0.52 V to 0 V. At the same time, an important feature revealed by the curves is an anodic oxidation peak at −0.2 V reveals that chromium oxide dissolution is taking place through the Cr^3+^ to the Cr^6+^ when the material suffers high anodic polarizations.

### 3.2 The stability of passive film of ferritic stainless steel

**Fig. 2:**
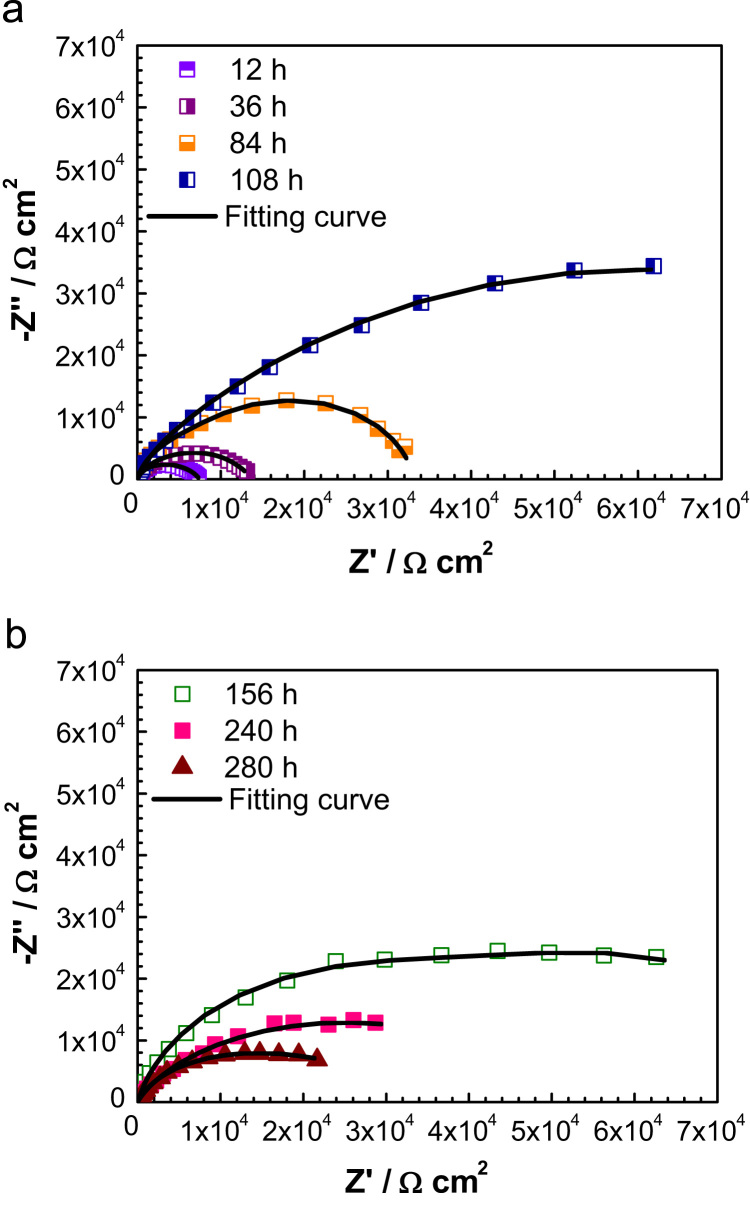
Nyquist plots of 430 ferritic stainless steel in alkaline solution with the presence of chloride ions after different immersion time.

Electrochemical impedance spectroscopy is very useful tools to study the stability of the stainless steel in different environments. Fig. 2 presents the open-circuit impedances spectra as a Nyquist diagram of 430 ferritic stainless steel at different immersion time into alkaline solution. It is clearly observed that all the electrochemical impedance plots with different immersion time were characterized by the presence of single unfinished semi-circle arc, which is attributed to charge transfer process occurring at the metal/electrolyte interface or related to the surface film property [2]. It is showing that they have the similar corrosion mechanisms. In the Nyquist diagram, the evolution of the overall impedance shows higher values with the immersion time increases, revealing an enhancement of passive film protective behavior in this type of environment. As can be seen in the Fig. 2(a), the global impedance increases during first immersion time, that is to say, with the immersion time from the first 12 h to 156 h, the overall impedance increase as the immersion increase. However, after the immersion time exceed 156 h, it slightly decreases, as can be seen in the Fig. 2(b), it revealing that the surfaces are more susceptible to pitting attack. From the above, the passive film of ferritic stainless steel formed in alkaline solution showed a similar evolution with time.

**Fig. 3:**
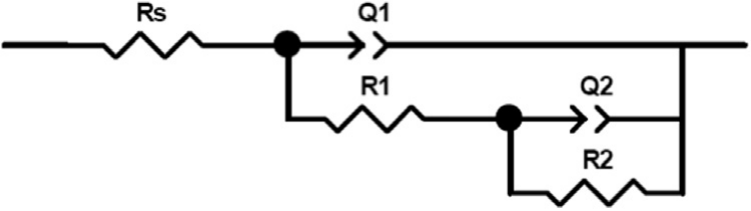
Equivalent circuit of the analysis of impedance spectra.

Fig. 3 exhibits the equivalent circuit of fitting EIS experimental results. Based on this model, it assumes that the passive film can׳t be considered as a homogeneous layer but rather as a defective layer. As is presented in Fig. 3, the equivalent circuit consists of the solution resistance *R*_s_ connected in series with two time constants *R*_1_[*Q*_1_(*R*_2_*Q*_2_)]. In the first process (*R*_1_*Q*_1_) at higher frequencies, the parameter *Q*_1_ represents capacitive behavior of the formed passive film, coupled with a resistance due to the ionic paths through the oxide film *R*_1_. In the second detected process, *Q*_2_ represents the capacitive behavior at the interfaces and *R*_2_ for the corresponding charge-transfer resistance.

**Fig. 4:**
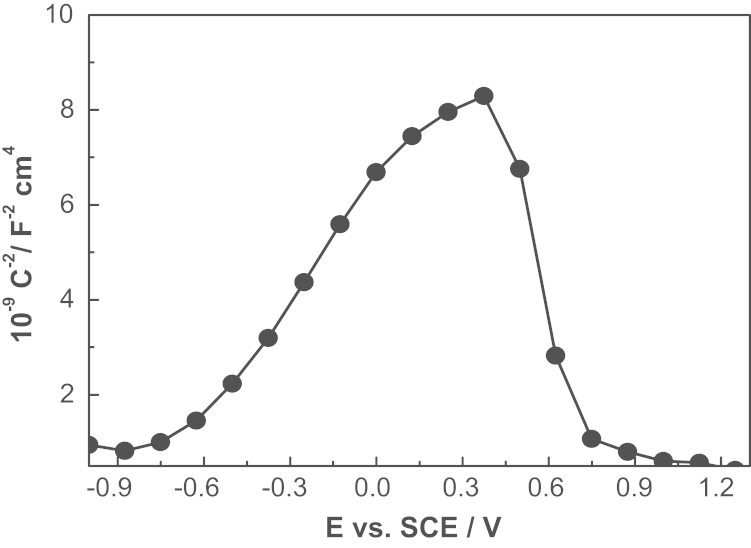
Mott–Schottky approach for passive film formed on the surface of ferritic stainless steel in alkaline solution at open circuit potential.

Fig. 4 shows the Mott–Schottky plots of 430 ferritic stainless steel in alkaline solution at the OCP condition as can be observed, the curves contained several regions, depending on the film formation potential, for the potentials above −0.8 V, a straight line with a positive slope, meaning that the oxides behaves as the n-type semiconductor in this potential region. As is known to all, the passive film of stainless steel can display both n-type and p-type behavior in different environments [3,4]. However, the shape of Mott–Schottky plot for the passive film formed on alkaline solution is far from that for the passive film on other conditions. The passive film of 430 ferritic stainless steel in the alkaline solution is close to the single layered Cr-substituted Fe-oxide rather than the duplex layered film of inner Cr oxide and outer Fe oxide. Moreover, there is a obviously change in the sign of the linear region slope at high potentials, going from positive to negative values. This phenomenon indicates a modification in the electronic properties of the passive film, from n-type to p-type semiconductivity, and is related to an increase in the conductivity of the film due to the solid state oxidation. The values of *N* doping density can be determined from the slope of the experimental *C*^−2^ vs. *E*. According to (1), the slopes of the linear portion of the *C*^−2^ vs. *E* give the charge carrier density *N*, from the relation [5]:

(1) $$N=\frac{2}{m\cdot e\cdot \varepsilon \cdot \varepsilon_{0}}$$

where *m* is the slope of the Mott–Schottky plot in the linear-region of interest, *e* is the electron charge, *ε* the relative dielectric constant of the semiconductor, *ε*_0_ the vacuum permittivity. According to the calculated results, the values of *N* are in the range of 10^20^−10^21^ cm^−3^, which are comparable to the reported values for stainless steel.

### 3.3 The character of passive film of ferritic stainless steel

#### 3.3.1 The thickness of passive film in simulated concrete pore media

**Fig. 5:**
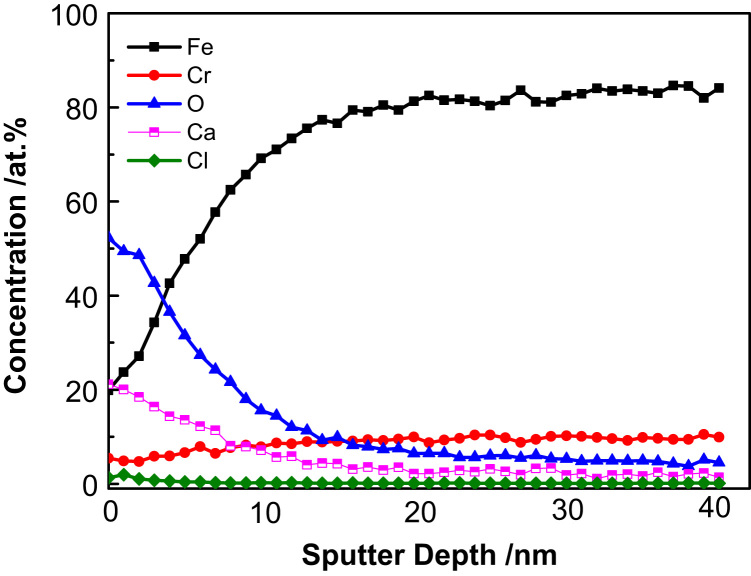
Auger depth profiles of the passive film formed at the surface in alkaline solution with the presence of chloride ions.

In order to perform a detailed investigation on the distribution of alloying elements in the passive film after immersion in alkaline solution, the depth profiles of passive film are examined by AES and the results are depicted in the Fig. 5. It is appeared that the Fe concentration in passive films is about 20–30 at% at the subsurface, and increased gradually with depth, and the Cr contents slightly increasing with the depth, but for the concentration profile of O rapidly decreases with prolonging the etching time. The boundary between the oxide layer and the bulk was never sharp, but an interface with a finite thickness is always observed. Methods developed by Sato and co-worker [6] allowed for the estimation of the interface position through extrapolation of the oxygen peak, for the boundary between the oxide layer and the bulk we always took a depth at which the oxygen concentration dropped to 50% of its maximum value. So, according to this, the thickness of passive film formed in alkaline solution is approximately 6 nm. As is known to all, the chloride attack is initiated by the adsorption of chloride ions on a metal surface, and then pitting corrosion occurred due to the local dissolution of this part. Fig. 4 illustrated the variation in the peak area of chloride in the passive film with etching time. It can be seen that the peak area of chloride in alkaline solution is slightly higher in the surface and then decreased to zero with the etching time, no chloride is observed in the passive film.

#### 3.3.2 The composition of passive film in simulated concrete pore media

**Fig. 6:**
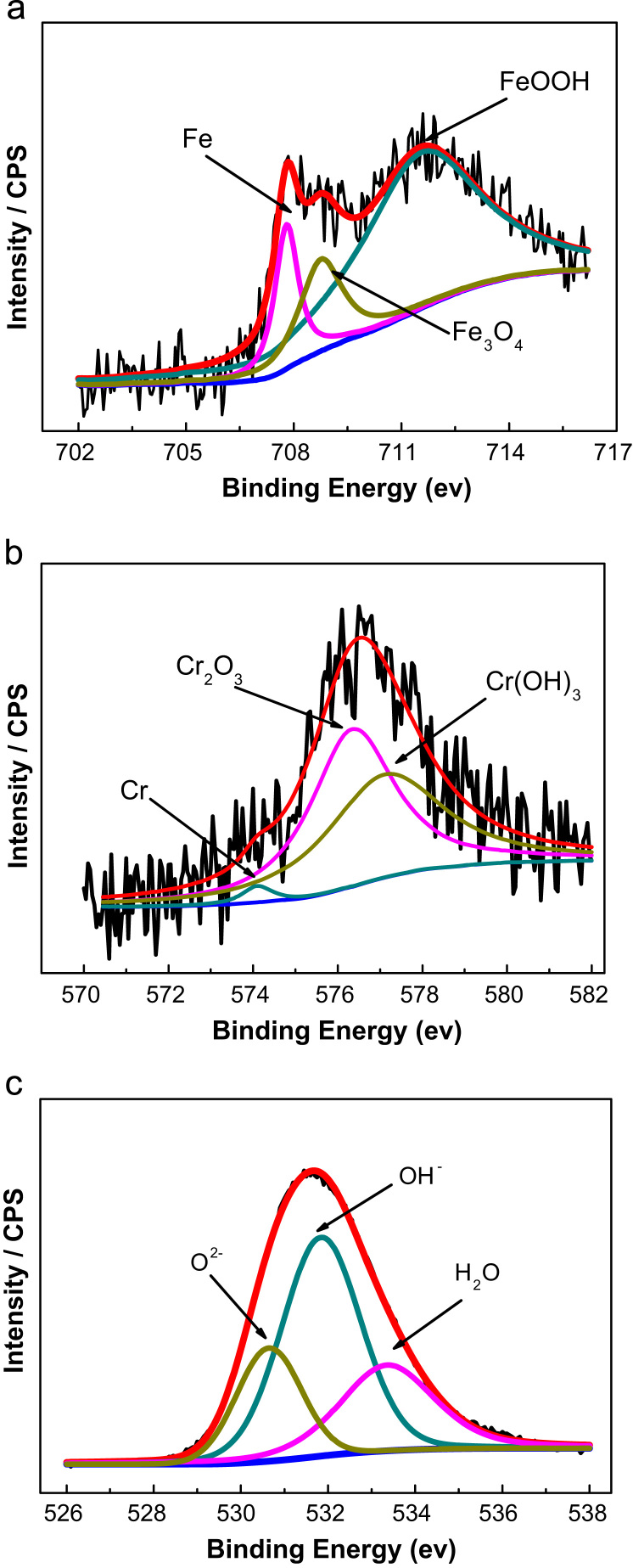
(a) The XPS spectra of Fe 2p_3/2_ of the passive films formed on ferritic stainless steel. (b) The XPS spectra of Cr 2p_3/2_ of the passive films formed on ferritic stainless steel. (c) The XPS spectra of O1s of the passive films formed on ferritic stainless steel.

Fig. 6 presented all of the metallic and oxidized states of Cr 2p_3/2_, Fe 2p_3/2_ and O1s. These results indicate that the primary constituents of the passive film are chromium oxide and iron oxide species, respectively [7]. As shown in the Fig. 6(a), Fe 2p_3/2_ spectra can be separated into several constituent peaks representing the metallic state (Fe_(met)_) (707.7 eV), the bivalent (Fe^2+^) and trivalent (Fe^3+^) species. The relative peak heights of FeOOH (711.8 eV) and Fe_3_O_4_ (708.2 eV) indicate they are the primary iron oxidized species in the passive film formed in alkaline condition. Fe_3_O_4_ is a mixed oxide composed by Fe^2+^ and Fe^3+^. It is known that the Fe^3+^ contribution from Fe_2_O_3_ and Fe_3_O_4_ is difficult to identify on XPS spectra [8] and a global value for the two iron oxides was considered. The formula for Fe_3_O_4_ may also be written as FeO·Fe_2_O_3_, showing one part wustite (FeO) and the other part hematite (Fe_2_O_3_). This refers to the different oxidation states of iron in crystalline structure, not in solid solution. Fe_3_O_4_ has a cubic inverse spinel structure which consists of a cubic close packed array of oxide ions where all the Fe^2+^ ions occupy half of the octahedral sites. The Fe^3+^ ions are split evenly across the remaining octahedral sites and the tetrahedral sites. Fig. 6(b) shows that there exist three constituent peaks representing metallic state Cr_(met)_ (574.1 eV), Cr_2_O_3_ (576.3 eV) and Cr (OH)_3_ (577.1 eV). The oxidized species are the primary constituents of the passive film. The intensities of the Cr_2_O_3_ states are apparently higher than that of the Cr (OH)_3_ and Cr_(met)_. The enrichment in Cr oxides improves the stability of the films. Oxygen species such as O^2−^ and OH^−^ in passive film, playing the role of connecting metal ions. Fig. 5(c) shows the core-level spectra of the passive film formed in alkaline environment in the O1s region. The O 1s spectra may also be split into three components O^2−^ (530.2 eV), OH^−^ (531.8 eV) and H_2_O (533 eV). It can be seen that OH^−^ is the primary constituent of the passive film, which corresponds to the formation of Cr(OH)_3_ and FeOOH. While O^2−^ is also the primary constituent of the passive film, which corresponds to the formation of Cr_2_O_3_, Fe_3_O_4_.
